# Preparation and in Vitro Property Research of Cholic Acid Nanoparticles with Dual-functions of Hemostasis and Antibacterial

**DOI:** 10.5812/ijpr-135437

**Published:** 2023-10-31

**Authors:** Jin Ma, Cong Wang, Tieying Yin, Yang Jiang, Wanjun Yu, Xiaoyu Zhang, Qin Qin, Hua Yang, Dechuan Zhang

**Affiliations:** 1 Department of Radiology, Chongqing Traditional Chinese Medicine Hospital, Chongqing, China; 2 Department of Ultrasonics, Chongqing Health Center for Women and Children, Women and Children's Hospital of Chongqing Medical University, Chongqing, China; 3 Key Laboratory for Biorheological Science and Technology of Ministry of Education, State and Local Joint Engineering Laboratory for Vascular Implants, Bioengineering College of Chongqing University, Chongqing, China

**Keywords:** Poly-l-lysine, Chenodeoxycholic Acid, Hemostasis, Antibacterial Effects

## Abstract

**Background:**

Hemorrhage control and anti-infection play a crucial role in promoting wound healing in trauma-related injuries.

**Objectives:**

This study aimed to prepare nanoparticles with dual functions of hemostasis and antibacterial properties.

**Methods:**

The dual-functional nanoparticles (CDCA-PLL NPs) were developed using a self-assembly method based on the electrostatic forces between poly-L-lysine (PLL) and Chenodeoxycholic acid (CDCA). The physicochemical properties, hemostatic properties, and antibacterial activities were investigated.

**Results:**

The prepared nanoparticles displayed a spherical structure, exhibiting a high drug loading capacity, encapsulation efficiency, and good stability. The CDCA-PLL NPs could reduce the hemolysis caused by PLL and promote the proliferation of human fibroblasts, indicating excellent biosafety. Moreover, CDCA-PLL NPs demonstrated a shorter in vivo hemostasis time and reduced blood loss in mouse tail vein hemorrhage, femoral vein hemorrhage, femoral artery hemorrhage, and liver hemorrhage models. Also, CDCA-PLL NPs showed excellent antibacterial efficacy against *E. coli* and *S. aureus*.

**Conclusions:**

CDCA-PLL NPs have great potential to be extensively applied as a hemostatic and antibacterial agent in various clinical conditions.

## 1. Background

Trauma-related injuries, especially the fatal bleeding caused by trauma, are the leading cause of death in young people (1-4). In most trauma cases, hemorrhage and infection simultaneously aggravate the injury. Nosocomial infections and massive bleeding remain the major causes of late mortality in trauma patients (5). Hence, hemostasis and anti-infection are important therapies in common trauma treatment (6, 7). When controlling bleeding, infection will affect the prognosis of wounds when infection control is ignored. In most occasions, hemostasis and anti-infection are applied separately, which leads to undesirable therapeutic effects, especially in some deep wounds (8). Traditional drug administration regimens involve the use of both anti-infective agents and hemostatic agents. However, this approach leads to a limited amount of anti-infective agents reaching the wound site, which in turn reduces the effectiveness of the drugs. Developing an advanced hemostatic formulation with dual functions of hemostasis and infection prevention is crucial for trauma-related injuries (9).

Poly-L-lysine (PLL) is a biocompatible, biodegradable, and water-soluble polypeptide; PLL and its derivatives are ideal for various biomedical applications (10-12). Poly-L-lysine was often used as nanocarriers to achieve high drug loading, targeted drug release, sustainable delivery control, and small side effects, among other benefits. In addition to being a carrier material, PLL has antibacterial effects (13-16). Since the biological membrane is often negatively charged, the positively charged PLL can trigger the easy adsorption of the formulation. Thus, PLL can enhance its cell interactions through electrostatic interactions and promote cell adhesion (17). Besides, PLL contains abundant cations; it can self-assemble into nanoparticles with drugs that contain anions through electrostatic forces (18, 19). Studies have also shown that PLL has hemostatic effects (20). It can neutralize the net negative charge on the platelet membrane and enhance platelet aggregation (21). The strong cationic characteristics can cause biological toxicity, but this problem can be solved by neutralizing them with an anionic material.

Bile acids are produced by the liver and can promote digestion and absorption of lipids (22). Bile acids have been proven to have antispasmodic, stomachic, hemostatic, and antibacterial effects (23, 24). Bile acids and their derivatives are organic acids; they all have a steroid structure and anionic carboxyl groups. They can act as amphipathic molecules and enhance permeation through biological membranes (25). Chenodeoxycholic acid (CDCA) is a primary bile acid commonly used to treat gall stones and lipid storage disorders (26). CDCA can be used to encapsulate lipophilic drugs and improve the stability of the drugs (27). The study incorporated CDCA into microcapsules containing lipophilic drugs, improving physical characteristics, stability, and release profiles (28). CDCA has also been proven to show some therapeutical effects, like reducing bacterial translocation (29) and controlling inflammation (30), contributing to the clinical potential of CDCA.

In this study, we employ PLL to prepare nanoparticles with chenodeoxycholic acid (CDCA), an anion-containing drug, expected that the nanoparticle would assemble spontaneously due to the coulombic interaction between the two components. At the same time, the anionic CDCA can neutralize the cation of PLL, resulting in better biosafety. To characterize the nanoparticles, we explored the morphology, particle size, zeta potential, drug loading capacity, encapsulation efficiency, and in vitro release profile. Different hemorrhage models were used to evaluate the hemostatic properties of CDCA-PLL NPs. Antibacterial effects of CDCA-PLL NPs were carried out on *S. aureus* and *E. coli*. The nanoparticles are speculated to have excellent hemostatic and antibacterial effects with good biocompatibility and low toxicity.

## 2. Objectives

This will provide a new approach for hemostasis and anti-infection in trauma treatment.

## 3. Methods

Chenodeoxycholic acid (CDCA, 99%) was purchased from Sigma Chemical Co., USA. DMEM culture medium was obtained from Gibco (Grand Island, NY, USA). Penicillin, streptomycin, and fetal bovine serum (FBS) were purchased from HyClone (Waltham, MA, USA). All the animal care and experimental protocols were performed in compliance with the Animal Management Rules of the Ministry of Health of the People’s Republic of China (No. 55, 2001) and the guidelines for the Care and Use of Laboratory Animals of the School of Pharmacy and Bioengineering, Chongqing University of Technology.

### 3.1. Preparation of Dual-functional Nanoparticles

Dual-functional nanoparticles were prepared by self-assembly method. Briefly, the predetermined weight of chenodeoxycholic acid (CDCA) and polylysine (PLL) were weighed and dissolved in 400 μL DMSO. After blending, the solution was added into a dialysis bag and dialyzed in deionized water for 8 hours. The deionized water was refreshed every 30 min, and the CDCA-PLL NPs were obtained.

### 3.2. Characterization of Dual-functional Nanoparticles

An appropriate amount of CDCA-PLL NPs suspension was applied to a copper grid, negatively stained, and placed under a transmission electron microscope (Tecnai, USA) to observe the microscopic morphology of the nanoparticles. The particle size and zeta potential analysis of the CDCA-PLL NPs were performed using a laser particle size analyzer (90Plus PALS, Brookhaven, USA). The stability of CDCA-PLL NPs was measured by monitoring the changes in particle size and zeta potential on the 0th, 1st, 2nd, 3rd, 4th, 5th, 6th, 7th, 30th, and 60th day. Fourier transform infrared spectroscopy (FT-IR) was used to confirm the formation of nanoparticles. The in vitro release profile of CDCA-PLL NPs was tested in PBS. Samples were taken at 0.5, 1, 1.5, 2, 4, 6, 8, 10, 12, 24, and 48 h, and released CDCA concentration was detected by HPLC.

### 3.3. Drug Loading Capacity and Encapsulation Efficiency

The drug content of CDCA-PLL NPs was measured by high-performance liquid chromatography (HPLC). The acetonitrile-0.1% phosphoric acid (45: 55) was used as the mobile phase, the detection wavelength is 192 nm, and the flow rate is 1 mL/min for the HPLC detection method of CDCA. Chenodeoxycholic acid was formulated into standard solutions at concentrations of 3 mg/mL, 2 mg/mL, 1 mg/mL, and 0.5 mg/mL. The solutions were filtered with a 0.22 μm organic filter injected into HPLC, and the standard curve was made according to the peak area and concentration. Chenodeoxycholic acid content was calculated according to the standard curve. The CDCA weight in CDCA-PLL NPs was calculated by dissolving the lyophilized CDCA-PLL NPs in methanol, injecting them into HPLC, and detecting them. The drug loading capacity and the encapsulation efficiency of CDCA-PLL NPs were calculated using the following formula:

Drug loading capacity % = m_t_ /m_0_×100

Encapsulation efficiency % = 𝑚_t_ / 𝑚_L_ × 100

Where m_0_ is the amount of CDCA-PLL NPs, m_t_ is the amount of CDCA detected in the system, and 𝑚_L_ is the total amount of materials added into the system.

### 3.4. Biosafety Evaluation

The biosafety properties of the formulation were estimated by hemolytic effects and cytotoxic effects of CDCA-PLL NPs. The hemolytic properties of CDCA-PLL NPs were evaluated using whole blood from mice.. 20 μL of deionized water, normal saline, CDCA-PLL NPs, CDCA, and PLL were added into the tubes, respectively. The deionized water was used as a positive control, and normal saline was used as a negative control. Whole mouse blood was collected, and 500 μl of it was added to the tubes. The solution was then mixed and centrifuged at 3000 r/min for 3 min. The supernatant was separated, and the hemoglobin concentration was measured using a microplate reader at 540 nm. The hemolysis rate of each sample was calculated according to the following formula.

Hemolysis rate= (OD_sample_ - OD_negative control_) / (OD_positive control _- OD_negative_ _control_) × 100%

To evaluate the safety and biocompatibility of CDCA-PLL NPs, human fibroblasts were incubated with CDCA-PLL NPs, and cytotoxicity was assessed by CCK8 assay. Human fibroblasts were cultured in a DMEM culture medium containing 10% FBS. 100 μL of cell suspension (1 × 10^4^ cells/mL) was seeded into 96-well plates and incubated at 37°C with 5% CO_2_ for 24 h. Poly-L-lysine, CDCA, and CDCA-PLL NPs were prepared into solutions containing 20 μg/mL, 40 μg/mL, and 80 μg /mL and added to the cell suspension. After 4 h incubation, the cells were washed twice with PBS, and a fresh cell culture medium containing 10% CCK-8 was added and incubated for 2 h at 37°C. The OD values of each cell were measured using an enzyme marker at a wavelength of 450 nm. The cell survival ratio was calculated using the following formula.

Cell survival ratio (%) = (As-Ab)/(Ac-Ab) × 100%

Where “As” refers to the OD values detected with cell suspension incubated with different drugs, “Ac” refers to the OD values of a control group without drugs, and “Ab” refers to the OD values of the blank group without cells or drugs.

### 3.5. In Vivo Hemostatic Properties of Dual-functional Nanoparticles

Since CDCA and PLL have shown potential for hemostasis, the hemostatic properties of CDCA-PLL NPs were evaluated using models of mouse tail vein hemorrhage, femoral vein hemorrhage, femoral artery hemorrhage, and liver hemorrhage. For the models of mouse tail vein hemorrhage, 24 male Kunming mice of 5 - 6 weeks, weighing 32 - 40 g, were randomly divided into four groups, including the control group treated with saline, PLL group, CDCA group, and CDCA-PLL group treated with CDCA-PLL NPs. The needles of the syringes were pierced into the tail vein at a 30-degree angle and slowly pulled out. The drugs were administrated immediately, and the timing was started until there was no obvious bleeding. During this process, the blood near the pinhole was gently sucked by the pre-weighted gauze strip. The blood loss was calculated by the gained weight of the gauze. For the mouse femoral vein hemorrhage model, the Kunming mice, weighing 32 - 40 g, were randomly divided into four groups. The animals were anesthetized and fixed, and the needles of the syringes were inserted into the femoral vein at a 45-degree angle and then slowly pulled out. The drugs were administrated immediately, and the timing was started until there was no obvious bleeding. The bleeding time and blood loss were recorded during the hemostatic process. The Kunming mice were randomly and equally divided into four groups for the mouse femoral artery hemorrhage model. The animals were anesthetized and fixed. The needles of the syringes were inserted into the femoral artery at a 45-degree angle and then slowly pulled out. The drugs were administrated immediately, and the timing was started until there was no obvious bleeding. The bleeding time and blood loss were recorded during the hemostatic process. For the mouse liver trauma hemorrhage model, the mice were anesthetized and fixed on a surgical corkboard. The liver of the mouse was exposed. The liver was placed on a pre-weighed filter paper, and a 5-mm-long and 2-mm-deep incision was made by a scalpel. Drugs were administrated, and the filter paper’s bleeding time and weight change were recorded as blood loss.

### 3.6. Influence on Platelet Aggregation

To further investigate the effect of hemostasis of CDCA-PLL NPs, we detected the influence of CDCA-PLL NPs on platelet aggregation. The orbital blood of Kunming mice was collected in an anticoagulation tube, centrifuged at 180 g for 10 min, and the upper platelet-rich plasma (PRP) was collected. 2 μl FITC- CD61 was added into 100 μL PRP and incubated for 40 min, and 20 μL of cy5.5-labeled CDCA-PLL NPs and PLL, unlabeled CDCA and saline were added into the solution, the system was incubated at 37°C for 20 min and observed with laser confocal microscope.

### 3.7. Antibacterial Capability of Dual-functional Nanoparticles

Hemorrhage and infection often coexist. Since PLL exhibits antibacterial function, the antibacterial effects of CDCA-PLL NPs on *S. aureus* and *E. coli* were further studied in this study. Briefly, 100 μL of *S. aureus* and *E. coli* solutions with 1 × 10^5^ CFU/mL concentration were added to a 96-well plate. Then, pure water, PLL, CDCA, and CDCA-PLL NPs were added and incubated at 37°C for 24 h. After incubation for 24 h, 100 μL of the bacterial broth was taken for plate coating, incubated at 37°C for 24 h, and then colony counting.

### 3.8. Statistical Analysis

Statistical analysis in this study was performed using Prism 8.2.0 (GraphPad Software, SanDiego, CA). A variance analysis (ANOVA) with two-tailed Student’s *t*-tests was used for experiments with independent continuous variables and more than two groups. All Data were expressed as mean ± standard deviation (SD), and significance was assessed when P < 0.05.

## 4. Results

### 4.1. Preparation and Characterization of Dual-functional Nanoparticles

**Figure 1.**
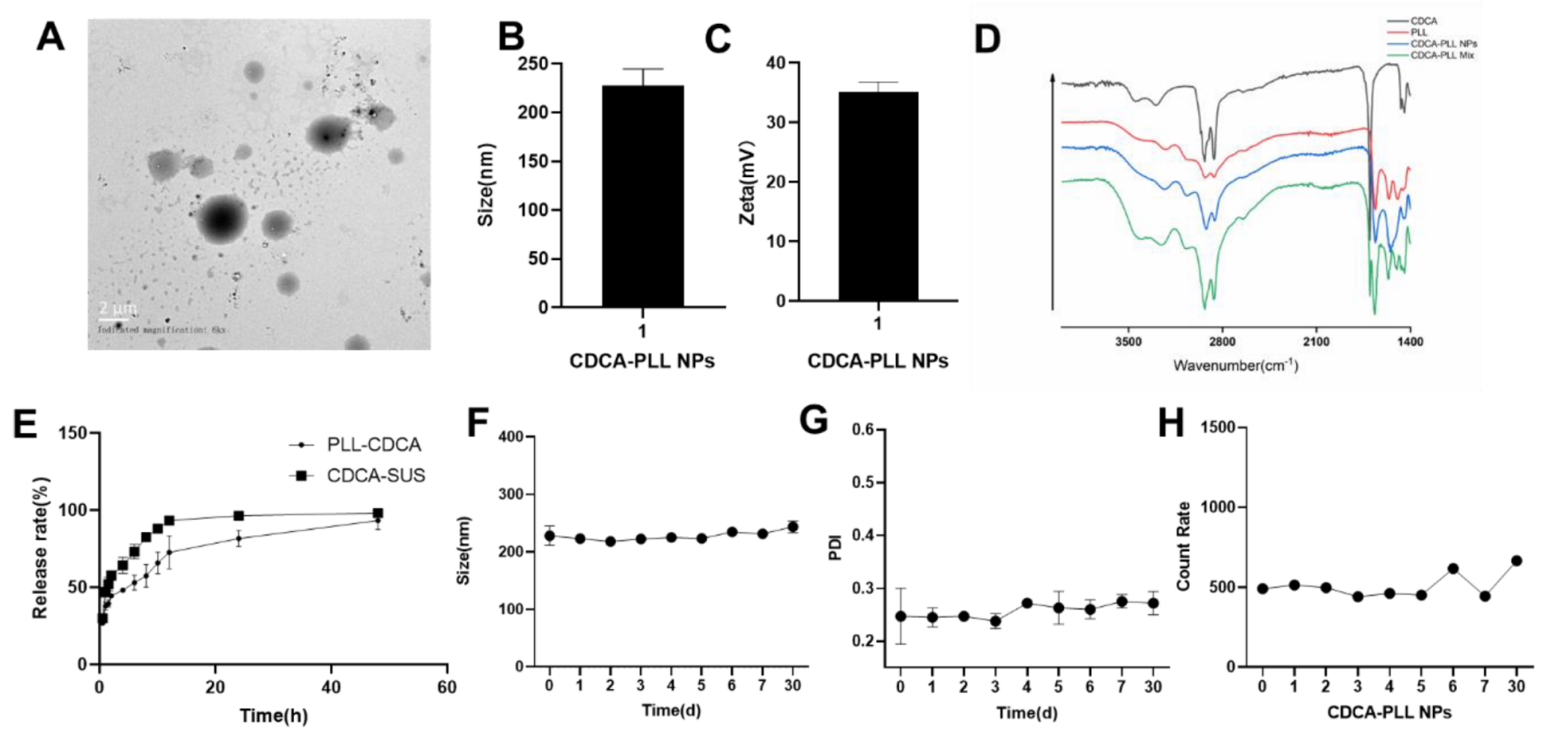
The characterization of dual-functional nanoparticles (CDCA-PLL NPs). A, the TEM image showed CDCA-PLL NPs were spherical granules; B, the particle size of CDCA-PLL NPs was about 227.7nm; C, the zeta potential of CDCA-PLL NPs was about 35.1 mv; D, FT-IR spectrogram of CDCA, PLL, CDCA-PLL NPs, and a mixture of CDCA and PLL proved the formation of nanoparticles; E, the in vitro release study of CDCA-PLL NPs indicated its slow release; F, the size change of CDCA-PLL NPs in 30 days; G, the PDI change of CDCA-PLL NPs in 30 days; H, the count rate change of CDCA-PLL NPs in 30 days.

The CDCA-PLL NPs were successfully prepared and characterized. The CDCA-PLL NPs were spherical granules (Figure 1A), the particle size of the NPs was 227.7±16.9 nm (Figure 1B), and the zeta potential was about 35.1±1.7 mv (Figure 1C). To investigate the stability of the NPs, we recorded the change in the size, PDI, and count rate of CDCA-PLL NPs. The stability results showed that the CDCA-PLL NPs had good stability within 30 days (Figure 1F, G and H). The formation of the nanoparticles may be due to the strong intermolecular interactions between CDCA and PLL, which could drive CDCA molecules to form amphipathic complexes with PLL and self-assemble into particles in aqueous. Fourier transform infrared spectroscopy showed the -NH_2_ of PLL and -OH of CDCA were both near 3302 cm^-1^. The C-H bond of PLL had tensile vibration at 2932cm^-1^, compared with a mixture of PLL and CDCA. Chenodeoxycholic acid-PLL NPs exerted shifts of the spectrogram, indicating the formation of nanoparticles (Figure 1D). In vitro release studies showed CDCA could be released from CDCA-PLL NPs with a slow-release profile (Figure 1E).

The drug loading capacity and encapsulation efficiency of CDCA-PLL NPs were detected by HPLC. We established the detection method of CDCA, and the standard curve equation was obtained as C=7.947·e^-007^ × A + 0.6889. According to the formula, the drug loading capacity of CDCA-PLL NPs was about 48.87%, and the encapsulation efficiency was about 73.3%, which was relatively high.

### 4.2. Biosafety Evaluation of Dual-functional Nanoparticles

**Figure 2.**
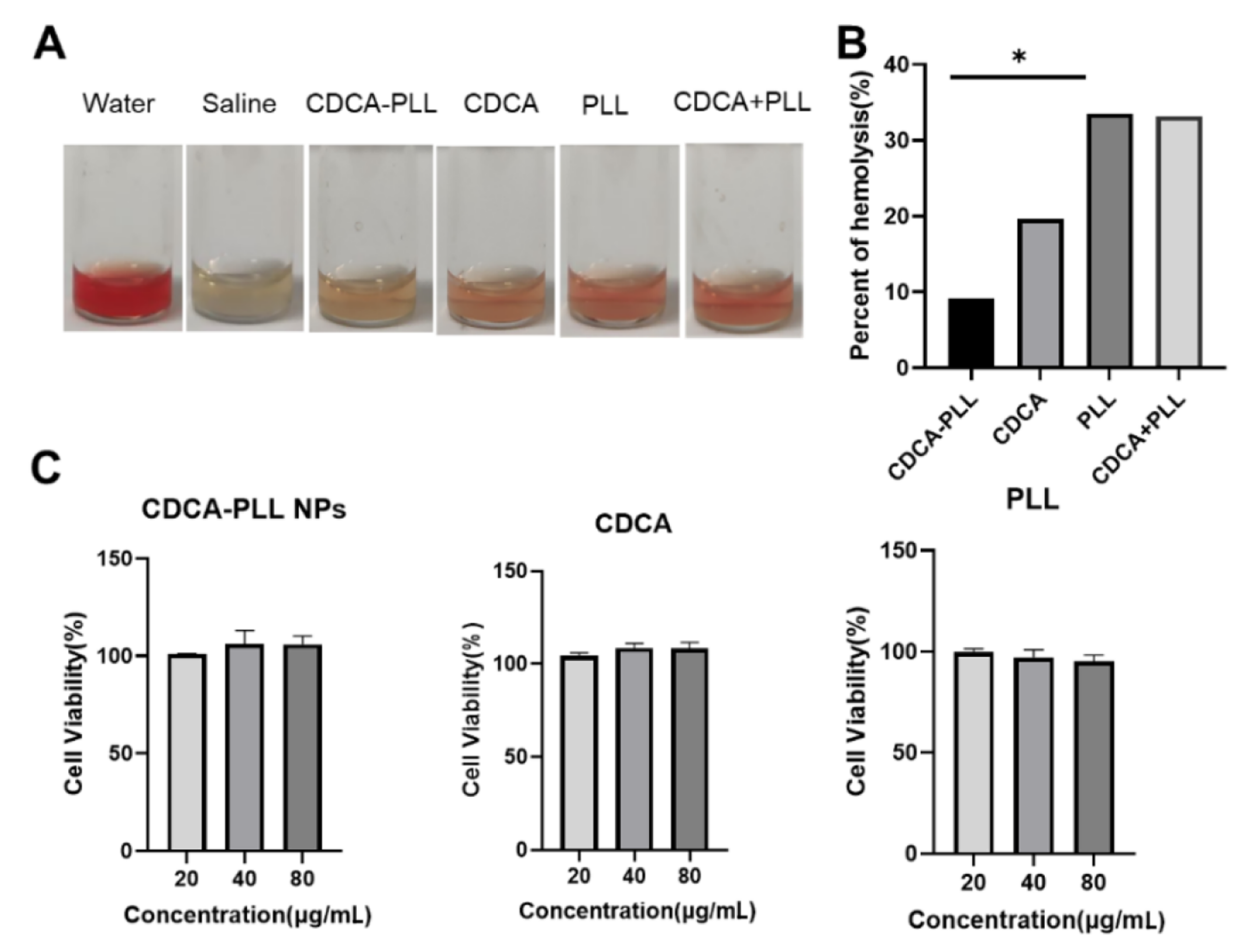
A, dual-functional nanoparticles (CDCA-PLL NPs) exhibit lower hemolytic properties compared to PLL; B, hemolysis percentage of different formulations; C, different formulations exhibit good biosafety with high cell viability. (* means P < 0.05)

Previous studies have shown that cholic acids exert procoagulant and antibacterial functions, which can also reduce the hemolysis of PLL. We detected the hemolytic properties of CDCA-PLL NPs. Compared with saline, water, PLL, and a mixture of PLL and CDCA showed marked hemolytic properties, but CDCA-PLL NPs could significantly reduce the hemolysis of PLL (Figure 2A and B).

In order to evaluate the safety and biocompatibility of CDCA-PLL NPs, we incubated CDCA-PLL NPs, CDCA, and PLL with human fibroblasts, respectively, and assessed the cell cytotoxicity of each solution. The results showed that each group exerted a high cell viability in the range of 20 - 80 μg/mL (Figure 2C), which indicated that CDCA-PLL NPs had no toxic effects on human fibroblasts. These results demonstrated that the combination of CDCA and PLL was safe and could even improve the biosafety of drugs.

### 4.3. In Vivo Hemostatic Performance of Dual-functional Nanoparticles

**Figure 3.**
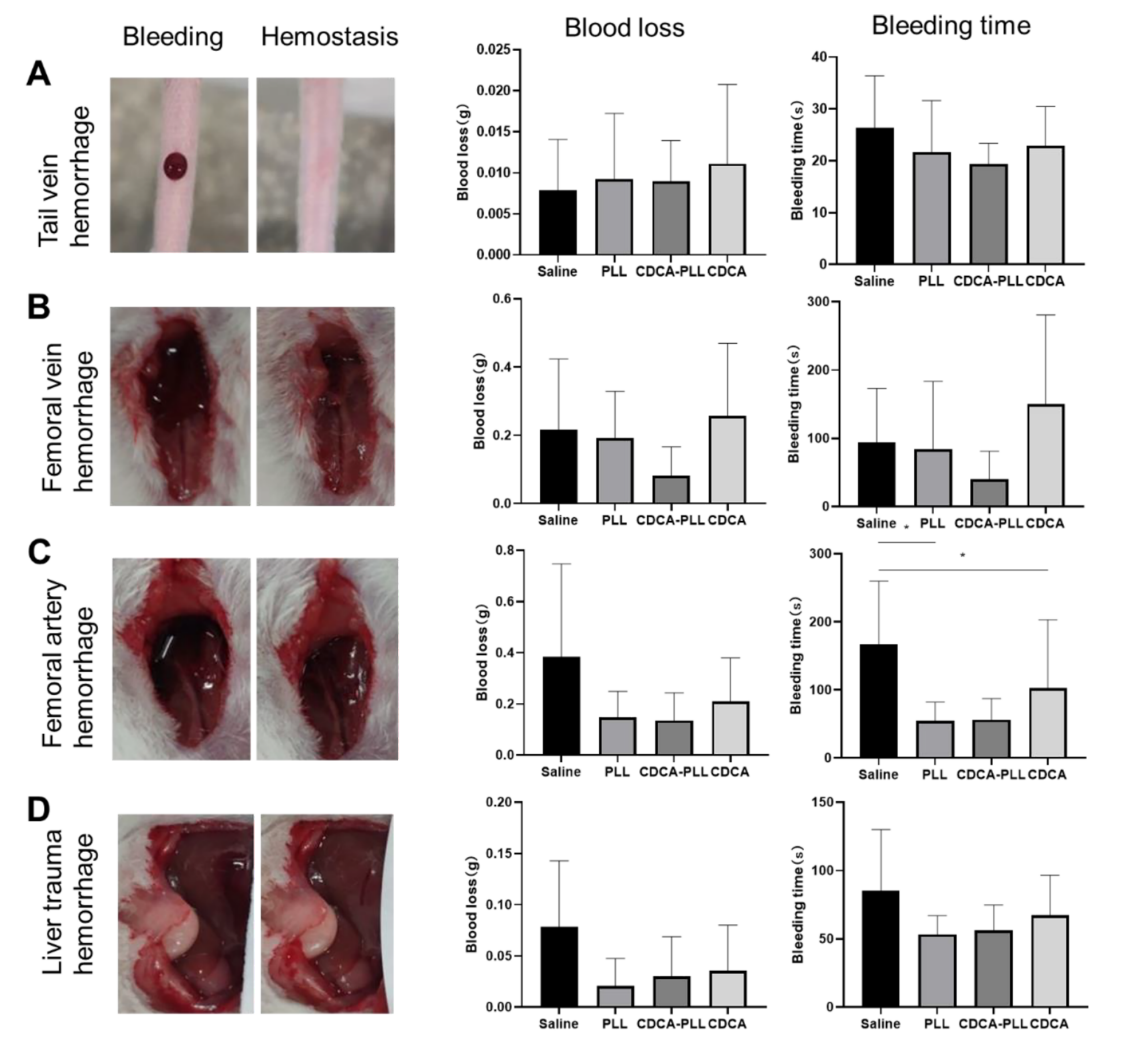
Hemostatic effects of topical administrations of dual-functional nanoparticles (CDCA-PLL NPs) in mouse tail vein hemorrhage model (A), femoral vein hemorrhage model (B), femoral artery hemorrhage model (C), and liver hemorrhage model (D). The left images represent several hemorrhage models, which showed the state of before and after bleeding. The right charts indicate bleeding time and blood loss, respectively. (* means P < 0.05)

Based on the obtained results, we further investigated the hemostatic performance of CDCA-PLL NPs in different bleeding models in vivo. The mouse tail vein hemorrhage model is often used as an in vitro trauma model, which can directly reveal the in vivo effects of coagulation drugs. In the mouse tail vein hemorrhage model, bleeding was easily observed after molding. After drug administration, the bleeding time of CDCA-PLL NPs was obviously shorter than that of normal saline (Figure 3A). 

The femoral vein and artery bleeding models represent deep vessel bleeding in vivo. In the femoral vein hemorrhage model, the bleeding time and blood loss were both significantly reduced compared with other groups (Figure 3B), while CDCA administration lengthened the bleeding time and increased the blood loss. In the femoral artery hemorrhage model, the bleeding time and blood loss were obviously reduced in the CDCA-PLL NPs and PLL groups (Figure 3C). The mouse liver hemorrhage model was used to simulate urgent internal severe hemorrhage and intraoperative hemorrhage. After modeling, a significant amount of blood was observed to be transudatory; CDCA-PLL NPs, CDCA, and PLL administration shortened the bleeding time and reduced the blood loss, among which CDCA-PLL NPs exerted the strongest effect (Figure 3D). In conclusion, CDCA-PLL NPs exhibited excellent hemostatic effects in multiple bleeding models in vivo.

### 4.4. Influence on Platelet Aggregation

**Figure 4.**
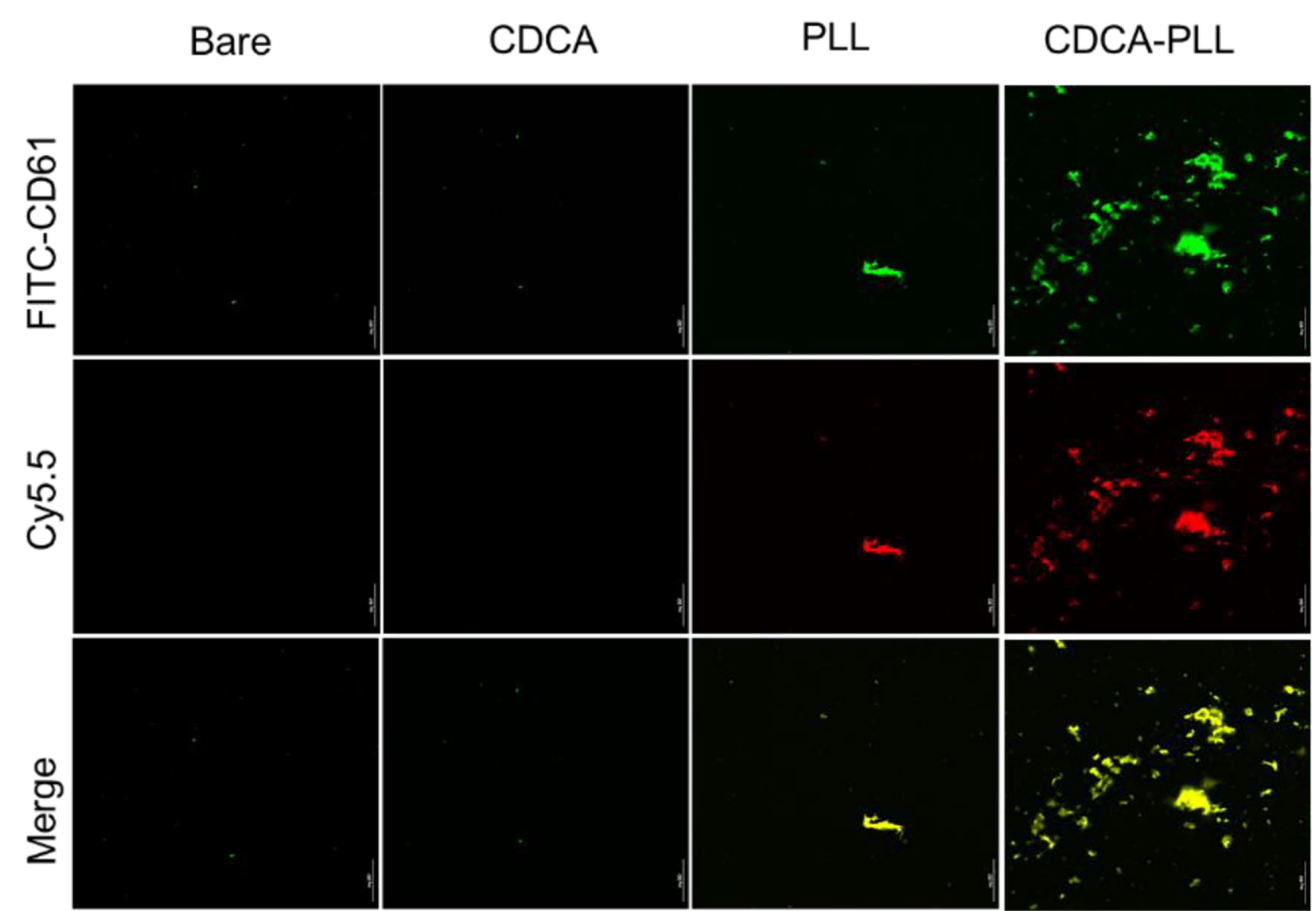
Laser confocal observation of platelet aggregation induced by different formulations, among which dual-functional nanoparticles (CDCA-PLL NPs) exerted the greatest effects

Based on the adhesion and activation of platelets caused by PLL and the hemostatic effects of CDCA-PLL NPs, we further detected the influence of CDCA-PLL NPs on platelet aggregation. The platelets were labeled with green fluorochrome, and the drugs were labeled with red fluorochrome; after incubating with PRP, PLL showed a marked platelet aggregation effect, while CDCA-PLL NPs exerted greater effects. The results indicated that the hemostatic effect of CDCA-PLL NPs might be achieved due to the effects of PLL and platelets (Figure 4). 

### 4.5. The Antibacterial Capability of Dual-functional Nanoparticles

**Figure 5.**
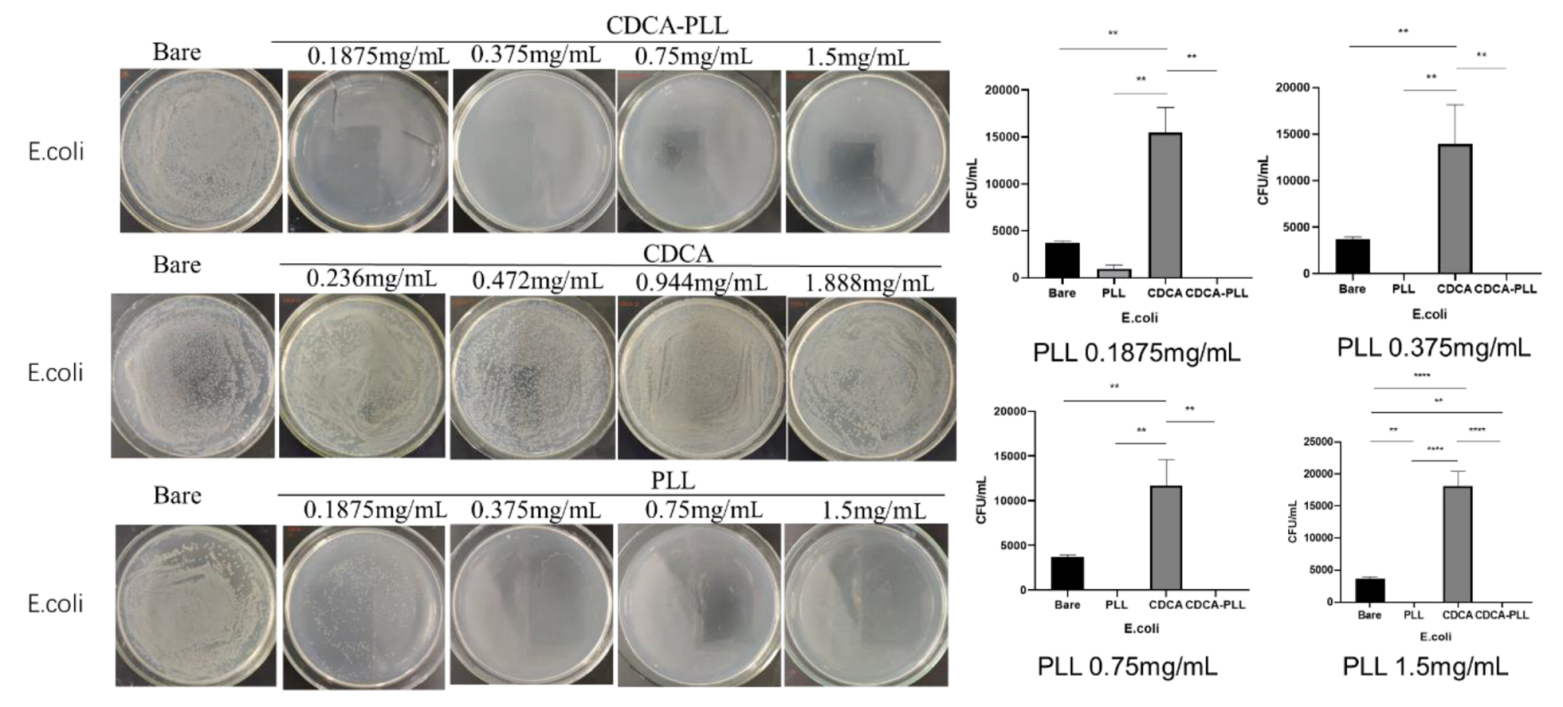
Antibacterial effects and colony counting results of CDCA-PLL NPs, CDCA, PLL against *E. coli*. (** means P < 0.05, **** means P < 0.0001)

Most hemorrhage cases, especially traumatic hemorrhage, are often accompanied by infection. Due to the antibacterial effect of PLL, we further explored the antibacterial capability of CDCA-PLL NPs. We determined that the minimum inhibitory concentration of PLL was 0.375 mg/mL against *E. coli* and 0.1875 mg/mL against *S. aureus* through the turbidity test. We used the different inhibitory concentrations of PLL to explore the antibacterial effects of CDCA-PLL NPs against *E. coli* and *S. aureus*. The research found that compared with the control group, PLL and CDCA-PLL NPs exerted excellent bacteriostatic effects against *E. coli* (Figure 5), among which CDCA-PLL NPs showed the best antibacterial effect, while CDCA showed little effect. In the bacteriostatic tests against *S. aureus*, we obtained similar results (Figure 5). The results showed that CDCA-PLL NPs inherited the excellent bacteriostatic effects of PLL.

**Figure 6.**
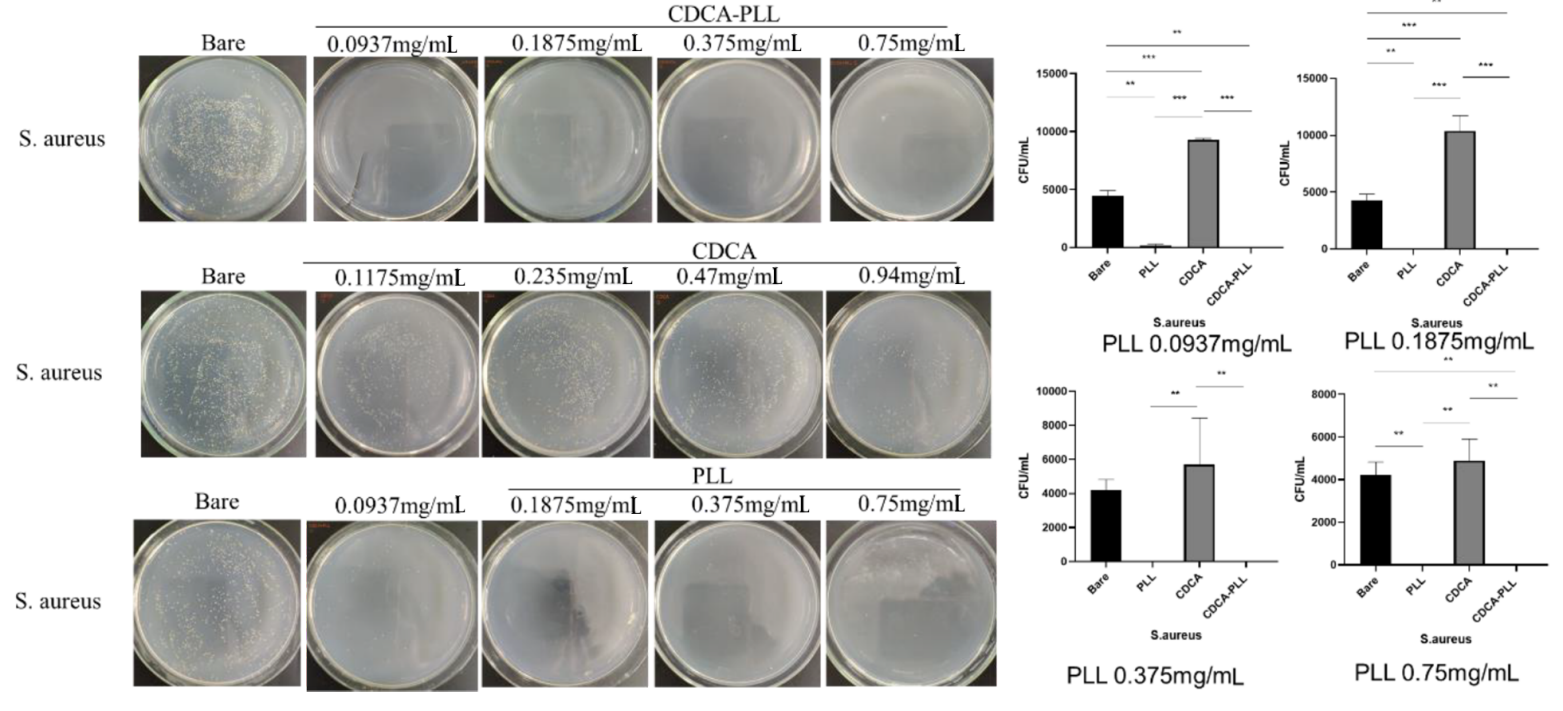
Antibacterial effects and colony counting results of CDCA-PLL NPs, CDCA, PLL against *S. aureus*. (** means P < 0.05, *** means P < 0.001)

The antibacterial mechanism of CDCA-PLL NPs may be due to the cationic polymer, PLL, which could interact with the bacterial cell membrane and destroy the membrane, leading to the death of bacteria (31). When the cationic PLL entered the bacteria, it could absorb negatively charged cells, destroying their energy metabolism, respiration, and electronic transmission systems (32, 33). Besides, PLL could affect intracellular ROS accumulation in bacteria, which also caused bacterial death (13) (Figure 6). 

## 5. Conclusions

We successfully prepared the CDCA-PLL NPs using a self-assembly method. The CDCA-PLL NPs obtained showed good drug loading capacity and stability. The biosafety evaluation of CDCA-PLL NPs demonstrated that the NPs could reduce the hemolysis of CDCA and PLL and showed good cell viability, which indicated that CDCA-PLL NPs are safe for both in vitro and in vivo applications. Chenodeoxycholic acid-PLL NPs exerted good hemostatic performance in various hemorrhage models. The hemostatic effect may be attributed to the activation of platelets. Meanwhile, CDCA-PLL NPs exhibited excellent antibacterial effects against *E. coli* and *S. aureus*, attributed to the antibacterial properties of PLL. In conclusion, our study confirms that CDCA-PLL NPs have significant potential therapeutic effects on hemorrhage and infection in traumatic injuries. This finding can expand the clinical application of nanoparticles and provide new treatment approaches for these clinical diseases. However, our study on hemostasis and antibacterial effects is relatively limited. Further research is needed to investigate the effects and mechanisms of nanoparticles. Meanwhile, considering the hemostatic and antibacterial effects of nanoparticles, CDCA-PLL NPs may also have an impact on other types of injuries, including trauma. This will be investigated in our future research.

## Data Availability

The dataset presented in the study is available on request from the corresponding author during submission or after publication.
